# Insights into the inner workings of transformer models for protein function prediction

**DOI:** 10.1093/bioinformatics/btae031

**Published:** 2024-01-19

**Authors:** Markus Wenzel, Erik Grüner, Nils Strodthoff

**Affiliations:** Department of Artificial Intelligence, Fraunhofer Institute for Telecommunications, Heinrich-Hertz-Institut, HHI, Einsteinufer 37, 10587 Berlin, Germany; Department of Artificial Intelligence, Fraunhofer Institute for Telecommunications, Heinrich-Hertz-Institut, HHI, Einsteinufer 37, 10587 Berlin, Germany; School VI - Medicine and Health Services, Carl von Ossietzky University of Oldenburg, Ammerländer Heerstr. 114-118, 26129 Oldenburg, Germany

## Abstract

**Motivation:**

We explored how explainable artificial intelligence (XAI) can help to shed light into the inner workings of neural networks for protein function prediction, by extending the widely used XAI method of integrated gradients such that latent representations inside of transformer models, which were finetuned to Gene Ontology term and Enzyme Commission number prediction, can be inspected too.

**Results:**

The approach enabled us to identify amino acids in the sequences that the transformers pay particular attention to, and to show that these relevant sequence parts reflect expectations from biology and chemistry, both in the embedding layer and inside of the model, where we identified transformer heads with a statistically significant correspondence of attribution maps with ground truth sequence annotations (e.g. transmembrane regions, active sites) across many proteins.

**Availability and Implementation:**

Source code can be accessed at https://github.com/markuswenzel/xai-proteins.

## 1 Introduction

### 1.1 Protein function prediction

#### 1.1.1 Proteins—constituents of life 

Proteins are versatile molecular machines, performing various tasks in basically all cells of every organism, and are modularly constructed from chains of amino acids. Inferring the function of a given protein merely from its amino acid sequence is a particularly interesting problem in bioinformatics research.

Function prediction can help to rapidly provide valuable pointers in the face of so far unfamiliar proteins of understudied species, such as of emerging pathogens. Moreover, it makes the analysis of large, unlabeled protein datasets possible, which becomes more and more relevant against the backdrop of the massive and evermore growing databases of unlabeled nucleic acid sequences, which again can be translated into amino acid sequences. Next-generation DNA sequencers can read the nucleic acid sequences present in a sample or specimen at decreasing costs (Mardis 2017, Shendure *et al.* 2017), much faster than experimenters can determine the function of the genes and corresponding proteins. Therefore, databases with genes and corresponding amino acid sequences grow much more rapidly than those of respective experimental gene and protein labels or annotations. Besides, gaining knowledge about the mapping between amino acid sequence and protein function can help to engineer proteins for dedicated purposes too (Alley *et al.* 2019, Yang *et al.* 2019, Ferruz *et al.* 2022, Hie and Yang 2022, Madani *et al.* 2023).

#### 1.1.2 Machine learning approaches

Machine learning approaches to protein function prediction can include inferring enzymatic function (Dalkiran *et al.* 2018, Li *et al.* 2018, Zou *et al.* 2019, Yu *et al.* 2023), Gene Ontology (GO) terms (Kulmanov *et al.* 2017, You *et al.* 2018a,b, 2021, Kulmanov and Hoehndorf 2019, 2022, Strodthoff *et al.* 2020, Littmann *et al.* 2021), protein–protein/–drug interaction, remote homology, stability, sub-cellular location, and other properties (Rao *et al.* 2019, Bepler and Berger 2021). For structure prediction, the objective is to infer how the amino acid sequence folds into the secondary (Zhang *et al.* 2018, Rives *et al.* 2021) and tertiary protein structure (Torrisi *et al.* 2020, AlQuraishi 2021, Jumper *et al.* 2021, Weissenow *et al.* 2022). Several of the prediction tasks can be approached as well by transferring labels from similar sequences obtained via multiple sequence alignment (MSA) (Buchfink *et al.* 2014, Gong *et al.* 2016). Protein prediction models are compared by the scientific community in systematic performance benchmarks, e.g. for function annotation (CAFA, Radivojac *et al.* 2013, Jiang *et al.* 2016, Zhou *et al.* 2019), for structure prediction (CASP, Kryshtafovych *et al.* 2019, 2021), or for several semi-supervised tasks (Rao *et al.* 2019, Fenoy *et al.* 2022). Machine learning methods are continuing to win ground in comparison to MSA-techniques with respect to performance, have a short inference time, and can process sequences from the so-called “dark proteome” too, where alignments are not possible (Perdigão *et al.* 2015, Rao *et al.* 2019, Lin *et al.* 2023).

### 1.2 Protein language modeling and transfer learning

#### 1.2.1 Relations to NLP

Amino acid sequences share some similarities with the sequences of letters and words occurring in written language, in particular with respect to the complex interrelationships between distant elements, which are arranged in one-dimensional chains. Thus, recent progress in research on natural language processing (NLP) employing language modeling in a transfer learning scheme (Howard and Ruder 2018) has driven forward protein function prediction too (e.g. Strodthoff *et al.* 2020).

#### 1.2.2 Self-supervised pretraining

Typically, a language model is first pretrained on large numbers of unlabeled sequences in an unsupervised fashion, e.g. by learning to predict masked tokens (cloze task) or the respective next token in the sequences (which is why this unsupervised approach is also dubbed self-supervised learning). In this way, the model learns useful representations of the sequence statistics (i.e. language). These statistics possibly arise because the amino acid chains need to be stable under physiological conditions and are subject to evolutionary pressure. The learned representations can be transferred to separate downstream tasks, where the pretrained model can be further finetuned in a supervised fashion on labeled data, which are usually available in smaller amounts, considering that sequence labeling by experimenters is costly and lengthy.

#### 1.2.3 Model architectures

Transformer models (Vaswani *et al.* 2017) making use of the attention mechanism (Niu *et al.* 2021), such as bidirectional encoder representations from transformers (BERT, Devlin *et al.* 2018) are currently prevailing architectures in NLP. Transformers have been recently applied to the study of amino acid sequences too, pushing the state of the art in the field of proteomics as well (Rao *et al.* 2019, 2021, Nambiar *et al.* 2020, Bepler and Berger 2021, Littmann *et al.* 2021, Rives *et al.* 2021, Brandes *et al.* 2022, Elnaggar *et al.* 2022, Fenoy *et al.* 2022, Unsal *et al.* 2022, Lin *et al.* 2023, Olenyi *et al.* 2023). Recurrent neural networks (RNNs) using long short term memory (LSTM) cells are another model architecture that is particularly suited to process sequential data. RNNs have been successfully employed to protein (Strodthoff *et al.* 2020) and peptide (Vielhaben *et al.* 2020) property prediction as well, within the scheme of language modeling combined with transfer learning, as sketched out above.

### 1.3 Explainable machine learning

#### 1.3.1 Need for explainability

Transformers and other modern deep learning models are notorious for having often millions and sometimes billions of trainable parameters, and it can be very difficult to interpret the decision making logic or strategy of such complex models. The research field of explainable machine learning (Lundberg and Lee 2017, Montavon *et al.* 2018, Arrieta *et al.* 2020, Tjoa and Guan 2020, Covert *et al.* 2021, Samek *et al.* 2021) aims at developing methods that enable humans to better interpret—or to a limited degree: understand—such “opaque,” complex models. In certain cases, it was demonstrated that the methods can even help to uncover flaws and unintended biases of the models, such as being mislead by spurious correlations in the data (Lapuschkin *et al.* 2019).

#### 1.3.2 Attribution methods

Attribution methods, such as integrated gradients (IG) (Sundararajan *et al.* 2017), layerwise-relevance propagation (Bach *et al.* 2015, Binder *et al.* 2016) or gradient-weighted class activation mapping (Selvaraju *et al.* 2017), make it possible to identify those features in the input space that the model apparently focuses on, because these features turn out to be particular relevant for the final classification decision of the model. Further examples of model explainability methods include probing classifiers (Belinkov 2022), testing with concept activation vectors (Kim *et al.* 2018), and studying the attention mechanism (Jain and Wallace 2019, Serrano and Smith 2019, Bai *et al.* 2021, Niu *et al.* 2021). Explainability methods have been employed in NLP too (Arras *et al.* 2019, Manning *et al.* 2020, Chefer *et al.* 2021, Pascual *et al.* 2021). Moreover, researchers have started to explore using explainability methods in the area of protein function prediction (Upmeier zu Belzen *et al.* 2019, Taujale *et al.* 2021, Vig *et al.* 2021, Hou *et al.* 2023, Vu *et al.* 2023, Zhou *et al.* 2023).

### 1.4 Contributions of the article

#### 1.4.1 Goal of the study

Building upon this previous research on the interpretation of protein classification models, we aimed at exploring how explainability methods can further help to gain insights into the inner workings of the now often huge neural networks, and proceeded as follows.

#### 1.4.2 Specific contributions

**Figure 1.**
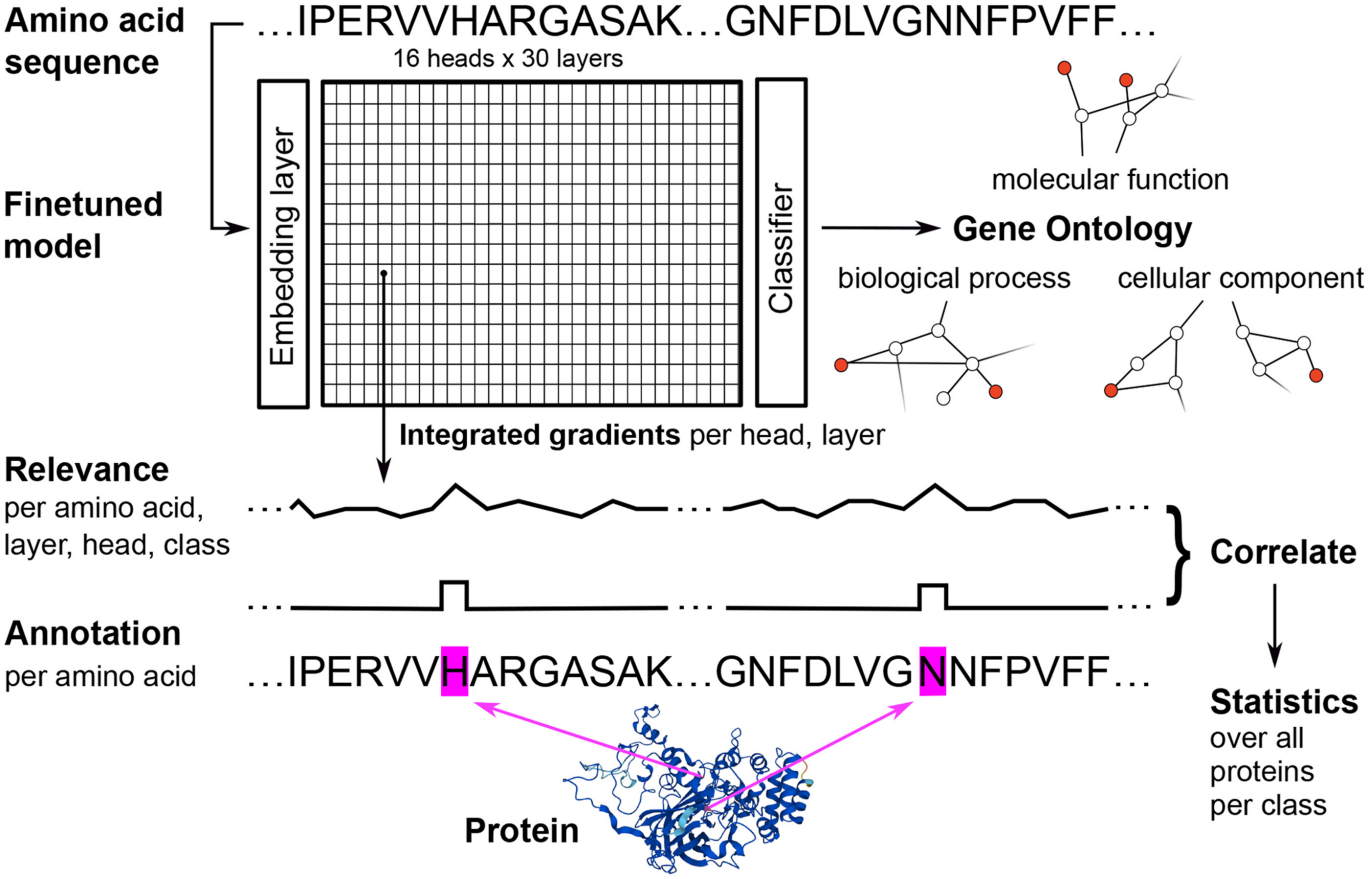
Illustration of the experimental design. Top: From the amino acid sequence, the finetuned transformer model infers the applicable Gene Ontology (GO) terms (represented as multi-label class membership vector). (The depicted exemplary “catalase-3” should be labeled with the GO terms “catalase activity” as “molecular function,” “response to hydrogen peroxide” as “biological process,” “cytoplasm” as “cellular component,” etc.; about 5K of about 45K GO terms were considered.) Center: Relevance indicative for a selected GO term was attributed to the amino acids per protein and correlated with corresponding annotations per amino acid. This correlation between relevance attributions and annotations was then statistically assessed across the test dataset proteins. The analysis was conducted for the embedding layer and “inside” of the model, for each head in each layer, and was repeated for different GO terms (see Section 2.1). Bottom: Specific amino acids of a protein are annotated in sequence databases like UniProt, because they serve as binding or active sites or are located in the cell membrane etc. Active sites can, e.g. be found at the histidine (“H” at position 65) and asparagine (“N” at position 138) of “catalase-3” (protein structure prediction created by AlphaFold—“AlphaFold Data Copyright (2022)DeepMind Technologies Limited”—under the CC-BY 4.0 licence (Jumper *et al.* 2021, Varadi *et al.* 2021).

First, we finetuned pretrained transformers on selected prediction tasks and could push or reach the state-of-the-art (see Supplementary Appendix E). Then, we quantified the relevance of each amino acid of a protein for the function prediction model. Subsequently, we investigated if these relevant sequence regions match expectations informed by knowledge from biology or chemistry, by correlating the relevance attributions with annotations from sequence databases (see Fig. 1). For instance, we addressed the question if a classification model that is able to infer if a protein is situated in the cell membrane does indeed focus systematically on transmembrane regions or not. We conducted this analysis on the embedding level and “inside” of the model with a novel adaptation of IG. In this way, we identified transformer heads with a statistically significant correspondence of the attribution maps with ground truth annotations, across many proteins and thus going beyond anecdotes of few selected cases.

## 2 System and methods

### 2.1 Revealing insights into function prediction models

#### 2.1.1 Prediction tasks

The prediction tasks of inferring GO terms and Enzyme Commission (EC) numbers, that the proteins are labeled with, from their amino acid sequence are detailed in Supplementary Appendix B. This Supplementary material also explains the finetuning of the transformers “ProtBert-BFD” and “ProtT5-XL-UniRef50” (Elnaggar *et al.* 2022) and “ESM-2” (Lin *et al.* 2023) on the GO and EC tasks, and contains statements about data availability and composition.

#### 2.1.2 Overall approach

We investigated whether specific positions or areas on the amino acid sequence that had been annotated in sequence data bases are particularly relevant for the classification decision of the model (see Fig. 1). Annotations included UniProtKB/Swiss-Prot “active” and “binding sites,” “transmembrane regions,” “short sequence motifs,” and PROSITE patterns related to a GO term and its children terms in the ontology. Definitions of the aforementioned UniProt annotations (per amino acid) and matching GO terms (class labels of proteins) are compiled in Supplementary Table A.1 (tables/figures with prefix letters are shown in the Supplementary material). First, we attributed relevance indicative for a given class (either a selected GO term or EC number) to each amino acid of a protein. Then, we correlated the relevance heat map obtained for the amino acid chain of a protein with corresponding binary sequence annotations. To study the information representation within the model, the explainability analysis was conducted at the embedding layer and repeated “inside” of the model, separately for its different heads and layers, using a novel method building upon IG, described below in Section 3.

#### 2.1.3 Experimental setup

For the experimental evaluation, we focus on the pretrained ProtBert model that was finetuned either to the multi-label GO-classification on the GO “2016” dataset, or to the multi-class EC number classification on the “EC50 level L1” dataset. We consider the comparatively narrow EC task in addition to the much more comprehensive GO prediction, because the test split of the EC dataset contains a larger number of samples that are both labeled per protein and annotated per amino acid, which is beneficial for the conducted explainability analysis. We observed that larger models tend to perform numerically better than smaller models (see Supplementary Appendix E). Given our focus on methodological matters of model interpretation, we deliberately studied ProtBert (420M parameters), because it is better manageable, due to its considerably smaller memory footprint, in comparison to the larger ProtT5 (1.2B).

## 3 Algorithm

### 3.1 Integrated gradients

Integrated gradients (Sundararajan *et al.* 2017) represents a model-agnostic attribution method, which can be characterized as unique attribution method satisfying a set of four axioms (Invariance, Sensitivity, Linearity, and Completeness). In this formalism, the attribution for feature *i* is defined via the line integral (along a path, parameterized as γ(t) with t∈[0,1], between some chosen baseline $\gamma (0)=x^{\prime }$ and the sample to be explained γ(1)=x),

(1) $${\text{IG}}_{i}^{\gamma }=\int_{0}^{1}d\alpha \frac{\partial F(\gamma (\alpha ))}{\partial \gamma_{i}}\frac{d\gamma_{i}}{d\alpha },$$

where *F* is the function we aim to explain. Choosing γ as straight line connecting $x^{\prime }$ and *x* makes IG the unique method satisfying the four axioms from above and an additional symmetry axiom. This path is the typical choice in applications applied directly to the input layer for computer vision or to the embedding layer for NLP. The approach can be generalized to arbitrary layers if one replaces *x* and $x^{\prime }$ by the hidden feature representation of the network up to this layer (referred to as “layer IG” in the popular “Captum” library (Kokhlikyan *et al.* 2020)).

### 3.2 Head-specific attribution maps

**Figure 2.**
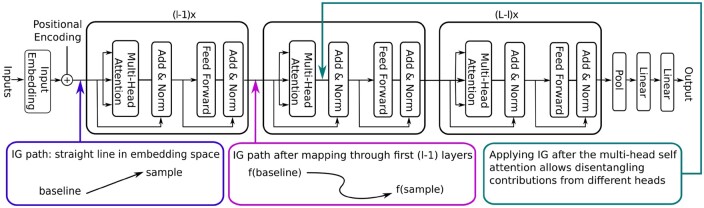
Visualization of the explainability method based on IG that can attribute relevance to sequence tokens (here: amino acids) separately for each head and layer of the transformer (adapted from Vaswani *et al.* 2017).

To obtain attributions for individual heads, we have to target the output of the multi-head self-attention (MHSA) block of a particular layer; see Fig. 2 for a visualization of the transformer architecture. Properly separating the attributions of the individual heads from the attribution contribution obtained from the skip connection necessitates to target directly the output of the MHSA. Now, one cannot just simply choose an integration path that connects baseline and sample as encoded by the MHSA block because the input for the skip connection has to be varied consistently. To keep an identical path in all cases, we fix the integration path as a straight line in the embedding layer, which then gets encoded into a, in general, curvilinear path seen as input for some intermediate layer. Choosing not a straight path only leads to the violation of the symmetry axiom, which is not of paramount practical importance in this application; see (Ward *et al.* 2020, Kapishnikov *et al.* 2021) for other applications with IG applied to general paths. For every sample, this application of IG yields a relevance map of shape $\text{seq}\times n_{\text{model}}$, where the first $n_{\text{model}}/n_{\text{heads}}$ entries in the last dimension correspond to the first head, followed by the second head etc. By summing over $n_{\text{model}}/n_{\text{heads}}$ entries in the last dimension, we can reduce the relevance map to a $\text{seq}\times n_{\text{heads}}$ attribution map, i.e. one relevance sequence per head.

### 3.3 Correlation coefficients and statistical significance

Each sequence of relevance attributions can then be correlated with sequence annotations to find out if the model focuses on the annotated amino acids. Coefficients of point biserial correlation (Kornbrot 2005), which is equivalent to Pearson correlation, were calculated between the continuous relevance values and the corresponding binary annotations per amino acid. This correlation analysis was conducted separately for each head in each transformer layer. The resulting correlation coefficients were then assembled into a $n_{\text{layer}}\times n_{\text{head}}$ matrix per protein, which entered the subsequent statistical analysis across proteins. Summary statistics over all proteins (which belong to the respective GO or EC class, and, which are part of the respective test dataset split) were obtained by computing Wilcoxon signed-rank tests across the correlation coefficients. The resulting *P*-values were corrected for the multiple tests per condition (16 heads times 30 layers equals 480 hypothesis tests) by controlling the false discovery rate (Benjamini and Hochberg 1995).

### 3.4 Summed attribution maps

In parallel to the correlation analysis, we furthermore sum the aforementioned attribution map along the sequence dimension, and obtain $n_{\text{heads}}$ entries that specify the relevance distribution onto the different heads. We can carry out the same procedure for every transformer layer and combine all results into a $n_{\text{layer}}\times n_{\text{head}}$ relevance map of summed attributions. This map makes it possible to identify heads with a positive relevance with respect to the selected class. One map was obtained per protein. Heads with a significantly positive relevance were singled out by calculating a summary statistic across proteins with the Wilcoxon signed-rank test. Finally, the two parallel analysis tracks were combined by identifying transformer heads that feature both a significantly positive (A) relevance-annotation-correlation and (B) relevance (this overlay is displayed in the figures by masking A with B).

## 4 Implementation

Supplementary Appendix D shows implementation details.

## 5 Results and discussion

### 5.1 Predictive performance

The performance results for the ProtT5, ProtBert, and ESM-2 transformers finetuned to the GO and EC protein function tasks are presented in Supplementary Tables E.1 to E.3 in Supplementary Appendix E. In summary, we show that finetuning pretrained large transformer models leads to competitive results, in particular in the most relevant comparison in the single-model category, often on par with MSA-approaches. Larger models lead the rankings, with ProtT5 competing with ESM-2. Finetuning the entire model including the encoder shows its particular strength in the “CAFA3” benchmark.

### 5.2 Explainability analysis: embedding layer

#### 5.2.1 Research question

Starting with embedding layer attribution maps, as the most widely considered type of attribution, we investigate whether there are significant correlations between attribution maps and sequence annotations from external sources (see Section 2.1). We aim to answer this question in a statistical fashion going beyond anecdotal evidence based on single examples, which can sometimes be encountered in the literature.

#### 5.2.2 GO prediction: GO “membrane” attributions correlate in particular with UniProt “transmembrane regions”

**Figure 3.**
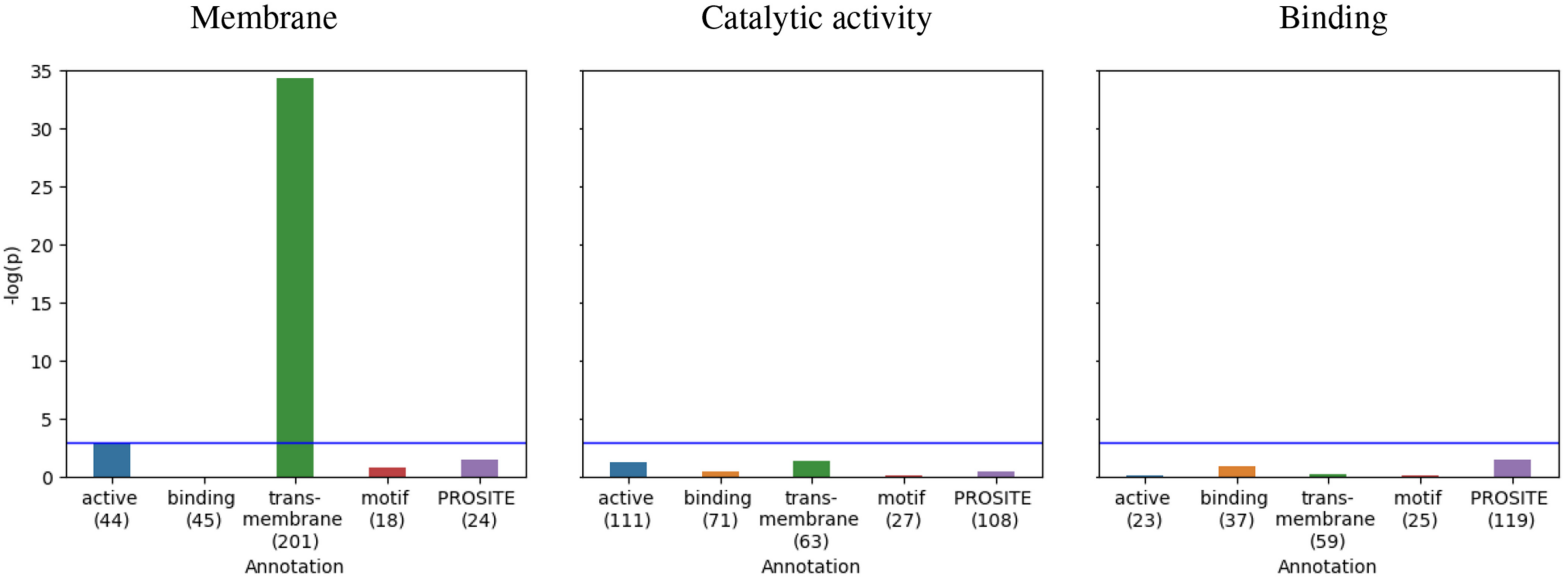
Attribution maps for the embedding layer of ProtBert finetuned to GO term classification were correlated with sequence annotations. Relevance attributions indicative for the GO label “membrane” correlate significantly with UniProt annotations as “transmembrane regions” (*p*<.05, i.e. above blue line). Attribution-annotation-correlation was not observed for GO “catalytic activity” and “binding.” Numbers of test split samples both labeled with the GO term and annotated per amino acid are listed below the *x*-axis.

Figure 3 shows the results of the explainability analysis for the embedding layer of ProtBert finetuned to GO classification. The relevance of each amino acid indicative for selected GO terms was computed with IG, and then correlated with UniProt and PROSITE sequence annotations. Subsequently, it was tested whether the correlation coefficients across all annotated proteins from the test set were significantly positive (see Section 2.1). A significant correlation was observed when relevance attributions indicative for the GO label “membrane” were correlated with UniProt “transmembrane regions” (*p*≪.05). Correlation was not observed in the GO “catalytic activity” and “binding” cases.

The pretrained model is expected to contain substantial information already prior to finetuning; e.g. Bernhofer and Rost (2022) had identified transmembrane regions using the pretrained ProtT5. Therefore, we inspected the GO membrane case in more detail. The pretrained but not finetuned ProtBert (combined with a classification head trained for the same number of epochs) resulted also in a significantly positive correlation of embedding level attributions to the GO term “membrane” with transmembrane regions only. Thus common patterns emerge between the pretrained and the finetuned ProtBert.

#### 5.2.3 EC prediction: attributions correlate significantly with several types of sequence annotations

**Figure 4.**
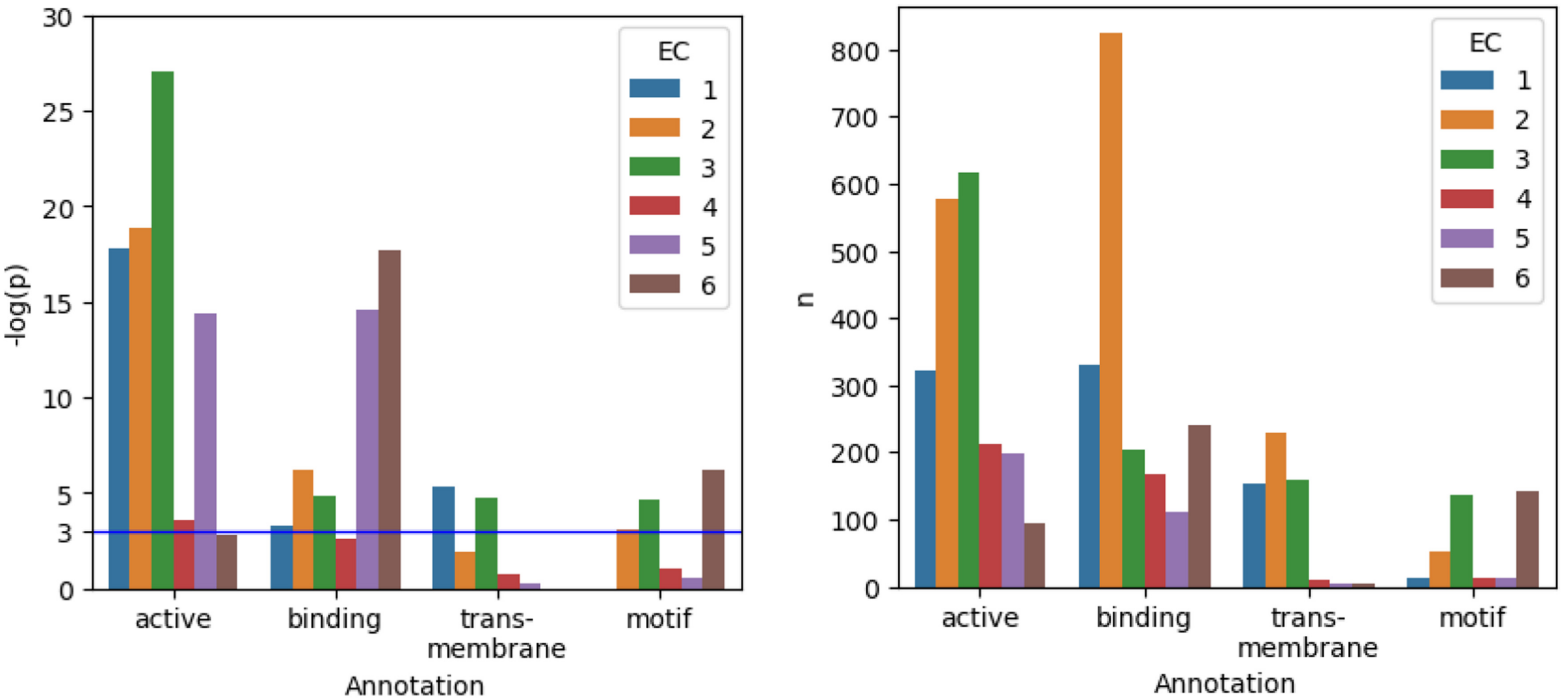
Attribution maps calculated for the embedding layer of ProtBert finetuned to “EC50 level L1” classification were correlated with UniProt sequence annotations. Left: Relevance attributions correlated significantly (*p*<.05, i.e. above blue line) with “active sites” and “binding sites” for five out of six EC classes, and with “transmembrane regions” and “short sequence motifs” for two, respectively, three EC classes each. Right: Numbers of annotated samples in the test split per annotation type and EC class.

Figure 4 shows the results of the explainability analysis for the embedding layer of ProtBert finetuned to EC number classification (“EC50 level L1” dataset; i.e. the differentiation between the six main enzyme classes). Relevance per amino acid for each of the six EC classes was correlated with the UniProt annotations as “active sites,” “binding sites,” “transmembrane regions,” and “short sequence motifs.” It can be observed that the relevance attributions correlated significantly (*p*<.05) with “active site” and “binding site” annotations for five out of six EC classes, and with “transmembrane regions” and “short sequence motifs” for two, respectively, three EC classes. (Supplementary Figure E.2 shows that positive relevance-annotation-correlation was observed for all annotation types for “EC40” and “EC50” on both levels “L1” and “L2” for several enzyme (sub-) classes.)

#### 5.2.4 Discussion

Attribution maps obtained for the embedding layer correlated with UniProt annotations on the amino acid level, in particular, in the EC case, but also for the GO term “membrane.” To summarize, across two tasks, we provide first quantitative evidence for the meaningfulness and specificity of attribution maps beyond anecdotal evidence. Note that the EC case has the benefit of often several hundred annotated samples contained in the test split (except for “transmembrane regions” and “motifs”; see right panel of Fig. 4). In comparison, the GO case provides fewer samples in the test split of the dataset that were also annotated on the amino acid level (see numbers in brackets below the *x*-axis in Fig. 3).

### 5.3 Explainability analysis: peeking inside the transformer

#### 5.3.1 Research question

Given the encouraging results presented in Section 5.2, we aim to go one step further and try to answer the more specific question if there are specialized heads inside of the model architecture for specific prediction tasks, using our IG variant that calculates relevance on the amino acid level per transformer head and layer (see Section 3).

#### 5.3.2 GO-prediction: membrane

**Figure 5.**
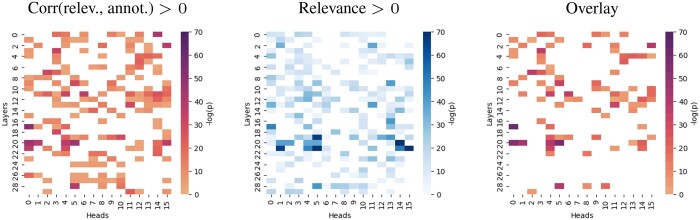
Inside ProtBert; GO “membrane” (GO:0016020). Left: The relevance attribution (along the sequences) indicative for the GO term “membrane” was correlated with UniProt annotations as “transmembrane regions,” for each transformer head and layer. Biserial correlation coefficients (*r*), obtained for each attribution-annotation-pair, were aggregated in population statistics with Wilcoxon signed-rank tests. The resulting *P*-values of the tests were adjusted with the Benjamini/Hochberg method for the multiple hypothesis tests conducted in order to limit the false discovery rate. A significance threshold was applied (family-wise error rate of 0.05). The negative logarithm of the corrected and thresholded *p*-values is displayed. All colored pixels indicate statistically significant results. Center: ProtBert heads with a sig. positive relevance (sum along the sequence; indicative for the GO term “membrane”) were singled out with the Wilcoxon signed-rank test. The matrix plots show the negative logarithm of the resulting *p*-values (adjusted with Benjamini/Hochberg and a threshold). Right: ProtBert heads with a sig. positive attribution-annotation-correlation (*p*-values from Wilcoxon signed-rank tests plotted) that are also characterized by a sig. positive relevance (the latter overlaid as mask). Only results for UniProt “transmembrane regions” are shown, omitting the results for “active/binding sites,” “motifs,” and PROSITE patterns, which did not feature heads with both a sig. positive relevance and attribution-annotation-correlation.

Figure 5 shows the results of the explainability analysis inspecting the latent representations inside of the ProtBert model focusing on the selected class of the GO term “membrane” (GO:0016020). Relevance attributions indicative for GO “membrane” per amino acid were correlated with the UniProt annotations as “transmembrane regions” separately for each transformer head and layer (matrix plot pixels in Fig. 5). In parallel, ProtBert heads were singled out with a significantly positive relevance (sum along the sequence) indicative for “membrane” (see also Section 2.1 and Section 3). Both parallel analysis streams were combined by identifying ProtBert heads with both a significantly positive attribution-annotation-correlation and relevance. Several ProtBert heads in different layers feature a significantly positive correlation of relevance attributions per amino acid with the UniProt annotations as “transmembrane regions,” going along with a significantly positive relevance for the GO class “membrane.” In contrast, correlation of relevance attributions with UniProt “active” or “binding sites” or “motifs” or PROSITE patterns accompanied by a positive relevance was not observed (hence these cases were not included in Fig. 5).

#### 5.3.3 GO prediction: catalytic activity

Supplementary Figure E.3 (in Supplementary Appendix E) shows the results of the explainability analysis for the case where the GO term “catalytic activity” was selected (GO:0003824). Different ProtBert heads stand out characterized by a positive relevance accompanied by a positive correlation of attributions with PROSITE patterns and with UniProt “active sites” and “transmembrane regions” (but neither with “binding sites” nor “motifs”).

#### 5.3.4 GO-prediction: binding

Supplementary Figure E.4 (in Supplementary Appendix E) repeats the explainability analysis inside ProtBert for the GO term “binding” (GO:0005488). For several transformer heads and layers, a positive relevance went along with a correlation of relevance attributions with corresponding PROSITE patterns, and with UniProt “transmembrane regions” (but neither with UniProt “active” nor “binding sites” nor “motifs”).

#### 5.3.5 EC-prediction

Subsequently, we conducted the explainability analysis for the case where ProtBert had been finetuned to EC number classification on EC50 level L1. Here, the model had learned to differentiate between the six main enzyme classes. Supplementary Figure E.5 (in Supplementary Appendix E) identifies ProtBert heads characterized both by a positive relevance (sum along the sequence) with respect to the EC class, and by a positive attribution-annotation-correlation (on the amino acid level). The analysis was conducted separately for UniProt annotations as “active”/“binding sites,” “transmembrane regions,” and “motifs.” (The absence of identified heads for EC4, EC5, and EC6 in the “transmembrane regions” rows and for EC1 and EC5 in the “motif” rows of Supplementary Figure E.5 goes along with the availability of relatively few “transmembrane” and “motif” annotations for these EC classes; see histogram in Fig. 4.)

#### 5.3.6 Discussion

In summary, we propose a constructive method suited to identify heads inside of the transformer architecture that are specialized for specific protein function or property prediction tasks. The proposed method comprises a novel adaptation of the explainable artificial intelligence (XAI) method of IG combined with a subsequent statistical analysis. We first attributed relevance to the single amino acids per protein (per GO term or EC class), separately for each transformer head and layer. Then, we inspected the correlation between relevance attributions and annotations, in a statistical analysis across the annotated proteins from the test split of the respective dataset. Apparently, different transformer heads are sensitive to different annotated and thus biologically and, respectively, chemically “meaningful” sites, regions or patterns on the amino acid sequence.

We discuss the benefits of finetuning a pretrained model from end-to-end, and evaluate the XAI method with a residue substitution experiment in Supplementary Appendix E. There, we also discuss the relation of XAI to homology, to the hydrophobicity and charge of residues in transmembrane regions, and to probing and in-silico mutagenesis.

### 5.4 Uncovering collective dynamics

**Figure 6.**
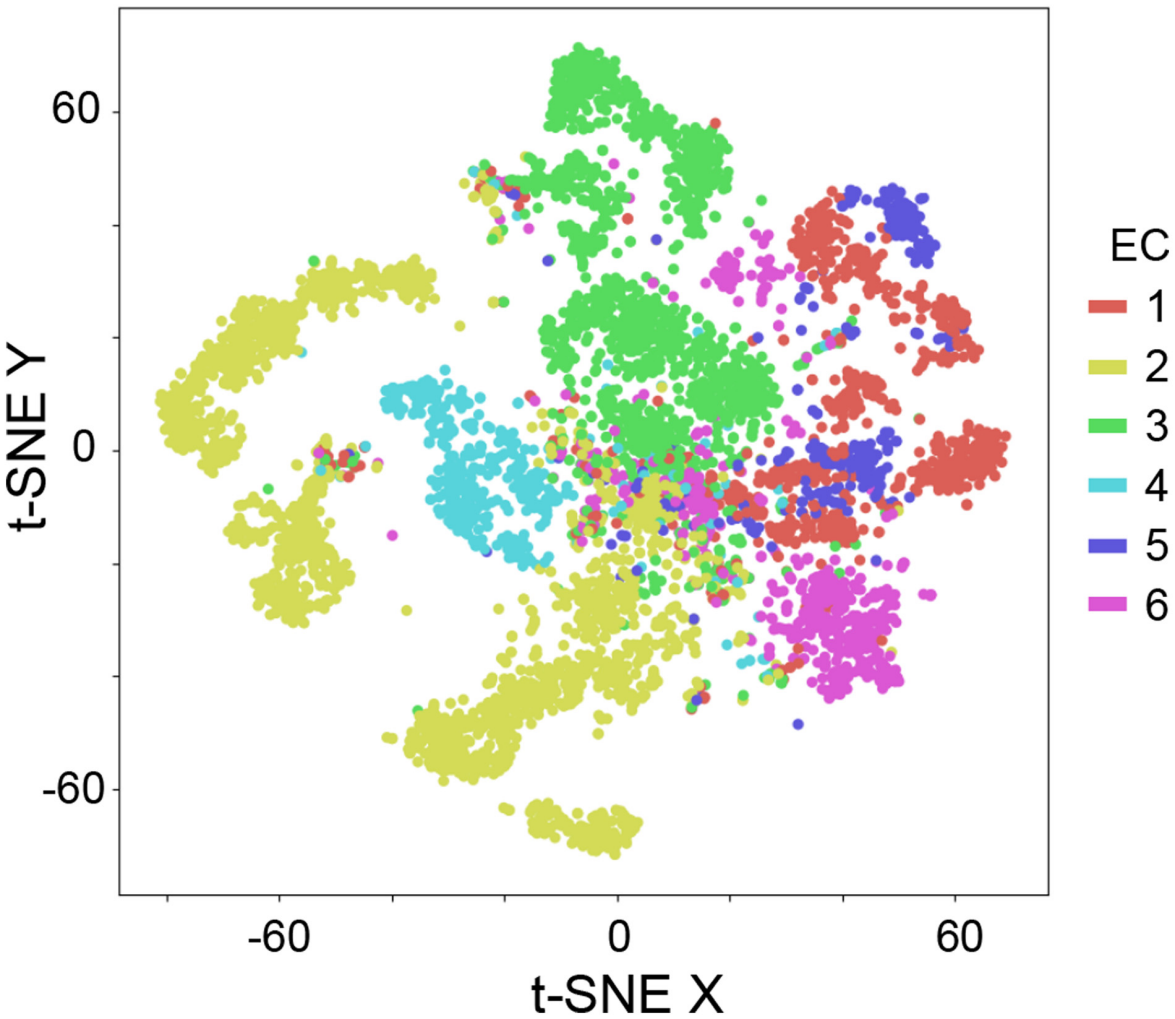
PCA and t-SNE visualization of summed attribution maps (ProtBert, EC50, L1).

Finally, we studied collective dynamics potentially emerging among the transformer heads (ProtBert, EC50, level L1) by a visualization of the originally high-dimensional, summed attribution maps in two dimensions, taking their similarities into account. For this purpose, the attribution maps that were summed along the amino acid sequence and represented as $n_{\text{layer}}\times n_{\text{head}}$ matrices (see Section 3) were flattened, resulting in one vector per protein. The dimensionality of these vectors was then reduced with principal component analysis to 50 dimensions, and subsequently to two dimensions with t-distributed stochastic neighbor embedding (t-SNE; van der Maaten and Hinton 2008), using the default t-SNE parameters. The resulting 2D points were visualized as scatter plot and colored according to the corresponding six main enzyme classes (Fig. 6).

The points form distinctive clusters matching the EC labels. Apparently, a structure emerges in the attribution maps, that seems to indicate class-specific collective dynamics among several ProtBert heads. It is important to stress that the attribution map underlying the clustering no longer contains any reference to specific positions in the sequence but relies on the relevance distribution on the different heads through all layers of the model. The emergence of class-specific structures therefore indicates that there are specific combinations of heads that are relevant for a specific classification decision.

## 6 Conclusion

This work provides additional evidence for the effectiveness of the currently predominant paradigm in deep-learning-based protein analysis through the finetuning of large protein language models from end-to-end (which brings additional benefits; see Supplementary Appendix E). For different protein function prediction tasks, this approach leads to best-performing models according to single-model performance. The performance level is in many cases on par with MSA-approaches. The proposed models can even be effectively combined with the latter through the formation of ensembles.

Considering the ever increasing model complexity, XAI has started to gain traction in the field of protein analysis too (Upmeier zu Belzen *et al.* 2019, Taujale *et al.* 2021, Vig *et al.* 2021, Hou *et al.* 2023, Vu *et al.* 2023, Zhou *et al.* 2023), but quantitative evidence for its applicability beyond single examples was lacking up to now. We provide statistical evidence for the alignment of attribution maps with corresponding sequence annotations, both on the embedding level as well as for specific heads inside of the model architecture, which led to the identification of specialized heads for specific protein function prediction tasks. Emerging class-specific structures suggest that these specialized transformer heads act jointly to decide together in specific combinations. A further detailed analysis of the identified heads could be an interesting next step in future research, potentially based on the query/key/value (QKV) matrices. Internally to the multi-layered model, a direct correspondence between rows/columns of the QKV matrices and individual residues in the sequence is, however, not possible anymore. This limitation makes it, e.g. difficult to infer relations between residues from the QKV matrices.

In summary, XAI promises to tap into the presumably substantial knowledge contained in large models pretrained on massive datasets of amino and/or nucleic acid sequences (Ji *et al.* 2021). Therefore, we expect that XAI will play an increasingly important role in the future of bioinformatics research. We see potential applications of XAI for model validation and for scientific discovery (e.g. of novel discriminative sequence patterns or motifs that have not been identified by experiments or MSA so far). Identifying specialized heads might also help to prune overly large models, making them smaller and more efficient.

## Supplementary Material

btae031_Supplementary_Data
