# Supplementary material for: Insights into the inner workings of transformer models for protein function prediction
Source: Bioinformatics. 2024 Jan 19;40(3):btae031. doi: 10.1093/bioinformatics/btae031 (PMC10950482; doi:10.1093/bioinformatics/btae031)
Supplement: btae031_Supplementary_Data [file btae031_supplementary_data.pdf]

A Supplementary material: Introduction

Table A.1. Definitions of UniProt annotations and of corresponding GO terms quoted from the UniProt and EMBL-EBI websites.

UniProt annotation (per amino acid)	GO term (per protein)
**Active site**: "used for enzymes and indicates the residues directly involved in catalysis"	**Catalytic activity** (GO:0003824): "Catalysis of a biochemical reaction [...] catalysts are naturally occurring macromolecular substances known as enzymes [that] possess specific binding sites for substrates."
**Binding site**: "interaction between protein residues and a chemical entity"	**Binding** (GO:0005488): "selective, non-covalent, often stoichiometric, interaction of a molecule with one or more specific sites on another molecule."
**Transmembrane region**: "extent of a membrane-spanning region of the protein. [...] both alpha-helical transmembrane regions and the membrane spanning regions of beta-barrel transmembrane proteins."	**Membrane** (GO:0016020): "A lipid bilayer along with all the proteins and protein complexes embedded in it an attached to it."
**Short sequence motif**: "a short (usually not more than 20 amino acids) conserved sequence motif of biological significance. Specific sequence motifs usually mediate a common function, such as protein-binding or targeting to a particular subcellular location, in a variety of proteins."	–

B Supplementary material: System and methods

I Protein function prediction tasks and data sets

GO prediction: introduction. We first focus on the protein function prediction task of inferring Gene Ontology (GO) terms, that the proteins are labeled with, from their amino acid sequence. This particularly comprehensive task allows characterizing each protein with respect to several properties, taking its molecular function, location in the cell, and involvement in different biological processes into account. GO term prediction has been approached using a range of deep learning methods in the past years (e.g. Kulmanov *et al.*, 2017; Kulmanov and Hoehndorf, 2019, 2022; You *et al.*, 2018b,a, 2021; Strodthoff *et al.*, 2020; Littmann *et al.*, 2021). The GO consortium (Ashburner *et al.*, 2000; Consortium, 2020a) aims at representing biological knowledge in the form of an ontology, i.e. as a graph comprising terms/classes as nodes and the relationships between them as edges. Actually, GO consists of three separate ontologies for molecular function (MFO), cellular component (CCO) and biological process (BPO). The protein database UniProtKB/Swiss-Prot (Consortium, 2020b) lists the amino acid sequences of proteins, and labels these proteins with selected, corresponding GO terms. In addition, the database contains selected annotations on the amino acid level, such as amino acids that serve as active or binding sites (see Table A.1), which will later play a role in this work.

GO prediction: setup. Considering that each protein can typically be labeled with several of the numerous GO terms, the task was phrased here as a multi-label ("protein-centric") prediction problem, where the model was trained to produce a label vector of about 5000 dimensions (5220 or 5101; see below) for each protein. The mapping from GO terms to the intricate topology of the ontology had been simplified by Kulmanov and Hoehndorf (2019), who made the data available in their accompanying data repository, as follows. A "flat" label vector represents the original graph structure of the ontology. Labels were propagated from children terms to parents through the hierarchy of the ontology (in contrast to the original GO, where labels are assigned to ontology leaves as far as possible). Only a subset of about 5k of the about 45k GO terms in total was considered, because the GO terms had been filtered to those that occur at least 50 times in the respective data set. Like in Kulmanov and Hoehndorf (2019), predictions are propagated towards the root node of the respective ontology tree. Evaluation is always performed with respect to the set of all GO terms, where the model can only predict the most frequently occurring GO terms.

GO prediction: data sets. We used both the "CAFA3" and the "2016" data sets by Kulmanov and Hoehndorf (2019) with 66841 training and 3328 testing samples with 5220 classes ("CAFA3") and, respectively, with 65028 training and 1788 testing samples with 5101 classes ("2016") (see Section 2.1 with Table 1 in Kulmanov and Hoehndorf, 2019). While the "CAFA3" data set is a widely used benchmark (Zhou *et al.*, 2019), the time-based "2016" splits of the underlying UniProtKB/Swiss-Prot database serve for the comparison with the results by You *et al.* (2018b,a); Kulmanov and Hoehndorf (2019); Strodthoff *et al.* (2020) ("2016" because entries from Jan.-Oct. 2016 serve for testing). The predictive performance of the protein function prediction models was assessed separately for the molecular function, cellular component and biological process ontologies of GO, with the $F_{\max}$ and $S_{\min}$ metrics (Clark and Radivojac, 2013) used in "CAFA3" (Zhou *et al.*, 2019), and with the area under the precision-recall (AUPR) curve (comparable to You *et al.*, 2018a; Kulmanov and Hoehndorf, 2019; You *et al.*, 2018b; Strodthoff *et al.*, 2020).

EC prediction. During the course of the present study, we narrow down the prediction task to the inference of the enzymatic function of a protein from its amino acid sequence. Enzymes are proteins that catalyze chemical reactions, which can be grouped into seven main classes and further sub-classes, as categorized by the Enzyme Commission (EC) numbers, a nomenclature developed by the International Union of Biochemistry and Molecular Biology (Webb *et al.*, 1992; McDonald *et al.*, 2008). EC prediction has been approached with machine learning, e.g. by Dalkiran *et al.* (2018); Li *et al.* (2018); Zou *et al.* (2019); Strodthoff *et al.* (2020); Yu *et al.* (2023).

The studied prediction task comprised the binary classification of whether a protein serves as enzyme or not (EC level "L0"), the classification into the main classes of enzymatic reactions (EC level "L1"), and the differentiation among different sub-classes of enzymatic reactions (EC level "L2"). We assessed the predictive performance on the "EC40" (58018 samples) and "EC50" (104940 samples) data sets by Strodthoff *et al.* (2020) (see their Supplementary Section S1), using accuracy as metric. The numbers in "EC40" and "EC50" refer to the similarity threshold between train and test splits of 40% similarity (more difficult task) and, respectively, 50% (easier task). The data set comprises the traditional six main EC classes (oxidoreductases, transferases, hydrolases, lyases, isomerases, ligases) leaving apart the new seventh EC class of translocases.

Data availability and composition of the training set. GO data by Kulmanov and Hoehndorf (2019) from their data repository and EC data by Strodthoff *et al.* (2020) (see their Supplementary Section S1) were pre-processed as detailed in the code repository by Strodthoff *et al.* (2020), resulting in separate data sets for GO "2016" (a.k.a. "temporalsplit"), for GO "CAFA3", and for EC40 and EC50 on levels L0, L1, and L2. Dedicated validation (a.k.a. development) splits were available for the EC and

GO “CAFA3” tasks. For the time-based GO “2016” split, a 10 % subset was randomly sampled from the training data and used as validation split (comparable to Kulmanov and Hoehndorf, 2019). As described below, we use the validation set for model selection and report the test set score.

II Finetuning pretrained transformers

Considered transformer models. We finetuned the transformer model “ProtBert-BFD” (Elnaggar *et al.*, 2022) to the GO term and EC number prediction tasks detailed earlier in Section I of Appendix B. “ProtBert-BFD” is a variant of BERT (Devlin *et al.*, 2018), with 420 million parameters distributed among 16 heads in 30 layers, that had been pretrained in a self-supervised way with a masked token prediction task on the massive metagenomic protein sequence collection titled “Big Fantastic Database” (BFD), which comprises more than two billion sequences (Jumper *et al.*, 2021; Steinegger *et al.*, 2019; Steinegger and Söding, 2018).

In addition, we finetuned also the more complex and memory intensive “ProtT5-XL-UniRef50” model (Elnaggar *et al.*, 2022) on the GO and EC tasks, using the last hidden state of the encoder. ‘ProtT5-XL-UniRef50” is based on the “Text-To-Text Transfer Transformer” (T5; Raffel *et al.*, 2020) with about three billion parameters (1.2 billion for the encoder used here) and had been pretrained on 45 million sequences from UniRef50 (Suzek *et al.*, 2014). We abbreviate “ProtBert-BFD” as “ProtBert” and “ProtT5-XL-UniRef50” as “ProtT5” in the following text.

Moreover, we finetuned two ESM-2 (Lin *et al.*, 2023) variants, both pretrained on UniRef50. The relatively small ESM-2 variant “esm2_t6_8M_UR50” comprises 6 layers, 8M parameters and an embedding dimension of 320. The larger variant “esm2_t33_650M_UR50D” (33 layers, 650M parameters, embedding dimension of 1280) was chosen, because its size is roughly comparable to ProtBert-BFD (30 layers, 420M parameters, 1024 encoder features) and ProtT5-XL-UniRef50 (24 layers, 1200M parameters, 1024 encoder features).

Classification head. The final classification head comprised a hidden layer and then a linear layer as connection to the dimensionality of the respective label vector. A hidden layer with $n = 50$ neurons was chosen for the simpler EC task. For the more demanding GO task, the size of the hidden layer was increased to $n = 2 \times 5220$ (GO “CAFA3”) and, respectively, to $n = 2 \times 5101$ (GO “2016”), such that the hidden layer contained twice as many neurons in comparison to the dimensionality of the respective label vector. Rectified linear units served as activation function, and layer normalization and a dropout layer with a dropout rate of ten percent served for regularization.

Finetuning procedure. For finetuning, the input sequence was cropped to a maximum sequence length of 1000 amino acids per protein, if necessary. The encoder features created by the transformer were pooled by concatenating the classification token, the maximum and the mean of the features along the sequence, and the sum of the features divided by the square root of the sequence length (Reimers and Gurevych, 2019). Binary cross entropy combined with a sigmoid layer served as loss function for the multi-label GO task, and cross entropy loss combined with softmax for the multi-class EC task. Gradients were accumulated over 64 batches of size one each. Adam (Kingma and Ba, 2015) was selected as optimizer. The learning rate was set to 5×10^{-6} for the encoder, which was frozen for the first epoch, and to 3×10^{-5} for the classification head. For the multi-label GO task, the rate was additionally scheduled with a linear increase over 500 warm-up steps in the beginning and then a decrease to zero following a half-cosine function calculated for a total number of 20000 training steps.

Model selection. After each training epoch, the loss (GO) or accuracy (EC) on the validation split was monitored and the model with the minimum loss (GO) or maximum accuracy (EC) was saved. Training was repeated until no further improvement was observed on the validation split. The best model was typically found after around 2-13 epochs in the case of ProtBert, after around 2-7 epochs in the case of ProtT5, after 6-20 epochs for the smaller, and 2-7 epochs for the larger ESM-2 variant.

C Supplementary material: Algorithm

Positive attributions. For the summed attribution maps (but not for the correlation analysis), the positive attributions are of higher importance, since this analysis focuses on identifying the areas of a sequence that speak for a specific class, rather than finding the areas that speak against this sequence being classified as such. Most XAI cases, such as the multiple classification attribution examples in Sundararajan *et al.* (2017), choose this approach. Some granularity is being lost because the attributions of individual amino acids are aggregated. The case that a head identifies the relevant areas with a positive attribution, and those values are canceled out by also finding negative-relevant areas is theoretically possible, even though a positive relevance sum is an indicator for the respective class, because the relevance sign is not arbitrary (Binder *et al.*, 2023). Nevertheless, we account for a potentially too rigid filtering by showing the correlation analysis results and the summed attribution maps individually.

D Supplementary material: Implementation

The pretrained models of ProtBert, ProtT5 (Elnaggar *et al.*, 2022), and ESM-2 (Lin *et al.*, 2023) were connected to an additional pooling layer and classification head (see Appendix B, II), and finetuned on GO data provided by (Kulmanov and Hoehndorf, 2019) and, respectively, EC data by Strodthoff *et al.* (2020) (see their Supplementary Section S1), using PyTorch (Paszke *et al.*, 2019) with PyTorch Lightning as wrapper. The adaptation of the integrated gradients algorithm acting separately on each transformer head in every layer (see Section 3) was implemented in Python building on the layer integrated gradients method of Captum (Kokhlikyan *et al.*, 2020). Annotations were obtained from the databases UniProt (Consortium, 2020b) and PROSITE (Sigrist *et al.*, 2012) in combination with GO (Ashburner *et al.*, 2000; Consortium, 2020a). Statistical calculations were performed using SciPy (Virtanen *et al.*, 2020) and statsmodels (Seabold and Perktold, 2010). Performance on the GO task was assessed based on an adaptation by (Strodthoff *et al.*, 2020) from (Kulmanov and Hoehndorf, 2019). Source code for model training, model interpretation, statistics and visualization can be accessed at `https://github.com/markuswenzel/xai-proteins`.

E Supplementary material: Results and Discussion

Research question. Here, we aim to answer the question whether finetuning of large pretrained transformer models leads to a competitive performance in protein function prediction.

GO prediction on the CAFA3 data set. Table E.1 shows the performance results for GO term prediction by finetuning and testing ProtBert, ProtT5 and ESM-2 on the “CAFA3” splits. Performance was assessed with the protein-centric $F_{\max}$ metric, separately for the molecular function (MFO), biological process (BPO), and cellular component ontologies (CCO) that comprise the GO. For comparison with the state-of-the-art, we added results reported in the literature (Olenyi *et al.*, 2023; Bernhofer *et al.*, 2021; Littmann *et al.*, 2021; Kulmanov and Hoehndorf, 2019; You *et al.*, 2018b) to Table E.1. For model evaluation, we distinguish between single-models and ensemble models. In the former category, the finetuned ProtT5 turns out to be the model that outperforms both prior deep-learning-based approaches based on transformers (goPredSim) and convolutional neural networks (DeepGOCNN) as well as the very strong MSA-based

Table E.1. GO term prediction by finetuning and testing ProtT5, ProtBert, and ESM-2 on the "CAFA3" splits, next to state-of-the-art results reported in the literature. Predictive performance was evaluated with the protein-centric $F_{\max}$ metric. The molecular function, biological process and cellular component ontologies of GO are abbreviated as MFO, BPO and CCO. Best single-model results are marked in bold face. Best overall results are underlined. Arrows (→) mark results achieved in this work. ProtT5 provides the best overall single-model performance in the MFO and CCO categories, while the larger ESM-2 variant performs best on BPO. Ensembling with the MSA-based DiamondScore predictions is highly effective.

	Method	MFO, $F_{\max}$ (($\uparrow$)	BPO, $F_{\max}$ ($\uparrow$)	CCO, $F_{\max}$ ($\uparrow$)
Single models	→ ProtT5-XL-UniRef50 (Elnaggar *et al.*, 2022), finetuned	**0.523**	0.442	<u>**0.641**</u>
	→ ProtBert-BFD (Elnaggar *et al.*, 2022), finetuned	0.503	0.441	0.630
	→ esm2_t33_650M_UR50D (Lin *et al.*, 2023), finetuned	0.512	**0.447**	0.639
	→ esm2_t6_8M_UR50D (Lin *et al.*, 2023), finetuned	0.503	0.419	0.629
	goPredSim, ProtT5 embeddings (Olenyi *et al.*, 2023)	**0.52**±3%	0.38±2%	0.59±2%
	goPredSim, SeqVec embeddings (Bernhofer *et al.*, 2021)	**0.52**±2%	0.37±2%	0.58±2%
	goPredSim, SeqVec embeddings (Littmann *et al.*, 2021)	0.50±3%	0.37±2%	0.57±2%
	DeepGOCNN (Kulmanov and Hoehndorf, 2019)	0.420	0.378	0.607
	DiamondScore (Kulmanov and Hoehndorf, 2019)	0.509	0.427	0.557
Ensembles	→ ProtT5-XL-UniRef50 (Elnaggar *et al.*, 2022), finetuned + DiamondScore	0.565	0.473	0.635
	→ ProtBert-BFD (Elnaggar *et al.*, 2022), finetuned + DiamondScore	0.580	<u>0.480</u>	0.640
	→ esm2_t33_650M_UR50D (Lin *et al.*, 2023), finetuned + DiamondScore	0.566	<u>0.480</u>	0.634
	→ esm2_t6_8M_UR50D (Lin *et al.*, 2023), finetuned + DiamondScore	0.559	0.470	0.629
	DeepGOPlus (Kulmanov and Hoehndorf, 2019)	0.544	0.469	0.623
	GOLabeler (You *et al.*, 2018b), (Fig. 3 of Zhou *et al.*, 2019)	<u>0.62</u>	0.40	0.61

baseline DiamondScore, on all ontologies MFO, BPO and CCO. The finetuned larger ESM-2 variant is a comparably strong competitor to ProtT5 (and better than ProtT5 on BPO).

Turning to ensemble model, we find that the ensemble of a finetuned ProtBert (followed by ProtT5) and DiamondScore (MSA-based; Kulmanov and Hoehndorf, 2019) produces the best predictions on CCO (scores were combined with a weighted sum; comparable to Kulmanov and Hoehndorf, 2019, see their Section 2.3). The ensemble of the finetuned larger ESM-2 variant with DiamondScore leads the ranking on BPO. The ensemble method "GOLabeler" (You *et al.*, 2018b) leads on MFO. GOLabeler had won the original CAFA3 challenge on all three ontologies BPO, CCO, and MFO (see Fig. 3 of Zhou *et al.*, 2019, where the $F_{\max}$ results are reported).

GO prediction on the 2016 data set. Table E.2 shows the GO term prediction results of the finetuned ProtT5, ProtBert, and ESM-2 models on the more recent "2016" data set Kulmanov and Hoehndorf (2019); You *et al.* (2018b), as well as the literature results (compiled by Kulmanov and Hoehndorf, 2019; Strodthoff *et al.*, 2020, which report AUPR and $S_{\min}$ results in addition to $F_{\max}$). Again, we distinguish between single and ensemble models in the following discussion.

Concerning single-models, different methods obtain the best results, depending on the considered metric and ontology. ProtT5 is the best single-model as measured by AUPR on all three ontologies MFO, BPO, CCO. ProtT5 also obtained the largest $F_{\max}$ on BPO while the larger ESM-2 variant is best on CCO. DiamondScore, an MSA-based method, is the best single-model with respect to the $S_{\min}$ metrics on the ontologies MFO and BPO, and achieves the best $F_{\max}$ on MFO. DeepGOPlus has the lowest and thus best $S_{\min}$ for CCO.

Turning to ensemble models, we find that the best overall results are obtained again by different ensemble methods. The finetuned ProtT5 combined with DiamondScore leads with respect to the AUPR metric and to $F_{\max}$ on BPO. The larger ESM-2 combined with DiamondScore leads the AUPR-ranking on CCO. Both ProtT5 and DeepGOPlus have the best $F_{\max}$ on CCO. DeepText2GO is best on MFO according to $F_{\max}$ and AUPR, and on CCO according to $S_{\min}$. GOLabeler features the smallest and thus best $S_{\min}$ on MFO and BPO.

EC prediction. Table E.3 shows how accurately ProtBert, ProtT5, and ESM-2 predicted the EC number of a given protein from its amino acid sequence, separately for the three EC levels (L0, L1, L2) and two train/test splits (EC40, EC50). For comparison, the table lists the results of "UDSMProt" too (Strodthoff *et al.*, 2020). The transformer models predict the EC number more accurately, with a large gap, in comparison to the RNN-based UDSMProt. ProtBert, ProtT5, and the larger ESM-2 variant are comparable with respect to their strong performance.

Discussion. Finetuning large, pretrained protein language models on the tasks of GO term as well as EC number prediction results in a strong predictive performance that is competitive with, and in several cases, better than state-of-the-art methods from the literature (depending on the metric and ontology under consideration, in the GO case). This outcome highlights again the benefits of transferring pretrained universal language models to downstream tasks in the proteomics field (cf., e.g. Strodthoff *et al.*, 2020; Rao *et al.*, 2019; Elnaggar *et al.*, 2022).

The largest inspected models lead the rankings on different metrics (and ontologies), with the bigger ESM-2 being a strong competitor to ProtT5. Again, ProtT5 consistently ranks better than (the smaller) ProtBert both on "CAFA3" and on the more recent, harder "2016" benchmark (only the ProtBert-DiamondScore ensemble forms an exception on "CAFA3" with respect to $F_{\max}$ and on "2016" with respect to $S_{\min}$ in the BPO case).

DeepGOCNN and DiamondScore are competing single-models with results available from the literature on both benchmarks. ProtT5, ProtBert, and ESM-2 outperform DeepGOCNN in both benchmarks on all metrics. ProtT5 and (in particular, the larger variant of) ESM-2 outperform the strong DiamondScore, which is based on MSAs, in both benchmarks in many cases.

The approach of finetuning the entire model including the encoder shows its strength in the "CAFA3" benchmark for BPO and CCO, where the finetuned ProtT5 outperforms the "goPredSim" model which is based on nearest-neighbor-lookup using features extracted from ProtT5 embeddings (Olenyi *et al.*, 2023). The observation that this nearest-neighbor-lookup is competitive in the MFO ontology might be related to the relatively dense annotation of the MFO.

Table E.2. GO term prediction by finetuning and testing ProtT5, ProtBert, and ESM-2 on the "2016" data set splits Kulmanov and Hoehndorf (2019); You et al. (2018b), next to the literature results collected by Kulmanov and Hoehndorf (2019) and Strodthoff et al. (2020). Best single-model results are marked in bold face (highest $F_{\max}$ and AUPR; lowest $S_{\min}$). Best overall results are underlined. Arrows (→) point towards models finetuned in this paper, with competitive results.

		$F_{\max}$ (↑)			$S_{\min}$ (↓)			AUPR (↑)		
	Method	MFO	BPO	CCO	MFO	BPO	CCO	MFO	BPO	CCO
Single-models	→ ProtT5-XL-UniRef50 (Elnaggar *et al.*, 2022), finetuned	0.526	**0.441**	0.685	10.099	35.229	8.030	**0.487**	**0.395**	**0.730**
	→ ProtBert-BFD (Elnaggar *et al.*, 2022), finetuned	0.503	0.424	0.677	10.421	35.401	8.198	0.460	0.369	0.714
	→ esm2_t33_650M_UR50D (Lin *et al.*, 2023), finetuned	0.507	0.433	**0.688**	10.256	35.291	8.124	0.466	0.376	0.726
	→ esm2_t6_8M_UR50D (Lin *et al.*, 2023), finetuned	0.520	0.423	0.681	10.016	35.618	8.199	0.486	0.371	0.715
	UDSMProt (Strodthoff *et al.*, 2020)	0.481	0.411	0.682	10.505	36.147	8.244	0.472	0.356	0.704
	DeepGO (Kulmanov *et al.*, 2017)	0.449	0.398	0.667	10.722	35.085	**7.861**	0.409	0.328	0.696
	DeepGOCNN (Kulmanov and Hoehndorf, 2019)	0.409	0.383	0.663	11.296	36.451	8.642	0.350	0.316	0.688
	DiamondScore (Kulmanov and Hoehndorf, 2019)	**0.548**	0.439	0.621	**8.736**	**34.060**	7.997	0.362	0.240	0.363
Ensembles	→ ProtT5-XL-UniRef50 (Elnaggar *et al.*, 2022), finetuned + ◇	0.591	<u>0.482</u>	<u>0.699</u>	8.583	33.752	7.467	0.571	<u>0.431</u>	0.736
	→ ProtBert-BFD (Elnaggar *et al.*, 2022), finetuned + ◇	0.583	0.470	0.693	8.687	33.560	7.597	0.558	0.423	0.725
	→ esm2_t33_650M_UR50D (Lin *et al.*, 2023), finetuned + ◇	0.594	0.476	0.697	8.656	33.498	7.583	0.564	0.427	<u>0.737</u>
	→ esm2_t6_8M_UR50D (Lin *et al.*, 2023), finetuned + ◇	0.598	0.476	0.696	8.600	33.576	7.542	0.567	0.424	0.729
	UDSMProt (Strodthoff *et al.*, 2020) + ◇	0.582	0.475	0.697	8.787	33.615	7.618	0.548	0.422	0.728
	DeepGOPlus (Kulmanov and Hoehndorf, 2019)	0.585	0.474	<u>0.699</u>	8.824	33.576	7.693	0.536	0.407	0.726
	DeepText2GO (You *et al.*, 2018a)	<u>0.627</u>	0.441	0.694	5.240	17.713	<u>4.531</u>	<u>0.605</u>	0.336	0.729
	GOLabeler (You *et al.*, 2018b)	0.580	0.370	0.687	<u>5.077</u>	<u>15.177</u>	5.518	0.546	0.225	0.700

◇: DiamondScore

Table E.3. The EC number of each protein was predicted from the amino acid sequence. Different EC levels (L0: enzyme or not, L1: six main enzyme classes, L2: enzyme sub-classes) and train/test splits (EC40/EC50) were assessed separately, with accuracy as performance metric. ProtT5, ProtBert and ESM-2 transformers were finetuned and the results compared to the RNN-based "UDSMProt" (cf. Strodthoff et al., 2020, Table 1; incl. baseline of a convolutional neural network (CNN) operating on MSA features). Arrows (→) mark results established here.

	EC40			EC50		
Model	L0	L1	L2	L0	L1	L2
→ ProtT5-XL-UniRef50 (Elnaggar *et al.*, 2022), finetuned	**0.966**	**0.968**	**0.954**	0.985	**0.993**	**0.985**
→ ProtBert-BFD (Elnaggar *et al.*, 2022), finetuned	0.960	0.958	0.945	0.981	0.990	0.982
→ esm2_t33_650M_UR50D (Lin *et al.*, 2023), finetuned	**0.966**	0.961	0.942	**0.986**	0.992	0.983
→ esm2_t6_8M_UR50D (Lin *et al.*, 2023), finetuned	0.926	0.855	0.811	0.958	0.943	0.929
UDSMProt ("Fwd+bwd; pretr.") (Strodthoff *et al.*, 2020)	0.91	0.87	0.84	0.96	0.97	0.95
CNN ("Baseline, seq") (Strodthoff *et al.*, 2020)	0.84	0.61	0.47	0.92	0.80	0.79

In summary, we showed that finetuning pretrained large transformer models leads to competitive results for protein function prediction tasks, in particular in the most relevant comparison in the single-model category.

On pretraining and finetuning from end-to-end. First of all, it is important to stress that our approach can only be applied to a model that includes a classification head as it relies on the class-specific output prediction. We studied how finetuning the entire model (comprising the pretrained ProtBert and the classification head) from end-to-end compares to training only the classification head while keeping the pretrained ProtBert frozen i.e. unchanged. We refer to these two scenarios as "finetuned" vs. 'pretrained'. We also considered an additional baseline where the ProtBert parameters were "shuffled" per layer, hence keeping realistic weight statistics across layers. Only the classification head was trained in the "shuffled" scenario, while the encoder parameters were kept frozen. For comparability, we trained the models in all scenarios for the same number of epochs, i.e. until early stopping was initiated in the "finetuned" scenario.

First, we inspected the performance results for these scenarios. For the GO term classification task "2016", the finetuned ProtBert (see Table E.2) achieved an $F_{\max}$ of 0.503 on MFO, of 0.424 on BPO, and of 0.677 on CCO. Performance dropped when only the classification head was trained, while the pretrained encoder was kept frozen, to an $F_{\max}$ of 0.463 on MFO, of 0.407 on BPO, and of 0.674 on CCO. Parameter shuffling resulted in an $F_{\max}$ of 0.305 on MFO, of 0.316 on BPO, and of 0.601 on CCO (close to the "naive" baseline from Table 3 of Strodthoff *et al.* (2020)).

For the enzyme classification task "EC50 level L1", the accuracy of 0.990 of the finetuned ProtBert, see Table E.3, dropped to an accuracy of 0.829 achieved by training only the classification head on top of the pretrained but frozen ProtBert. Performance dropped further to 0.369 in the "shuffled" scenario.

Thus, finetuning from end-to-end apparently provides advantages in comparison to only training the classification head on top of the pretrained encoder. Using pretrained parameters in both the "finetuned" and the "pretrained" scenarios shows the well-known benefits of transfer learning in comparison to the "shuffled" scenario.

The corresponding attribution analysis results (see Figure E.1) for the "finetuned" and the "pretrained" scenario have much in common – in contrast to the "shuffled" scenario, which shows a disparate picture. Overlap of "significant heads" between each scenario pair was quantified with the Jaccard similarity coefficient as 0.43 for the finetuned-pretrained pair, in contrast to the smaller 0.036 for the finetuned-shuffled pair, and 0.035 for the pretrained-shuffled pair. Both "finetuned" and "pretrained" scenarios

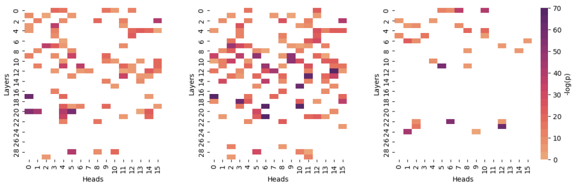

Figure E.1. Attribution analysis results were similar in the comparable “finetuned” (left) and “pretrained” (centre) scenarios, but different in the disparate “shuffled” (right) scenario, as expected (inside ProtBert, GO membrane/transmembrane). Jaccard similarity of the overlap of “significant heads” between scenarios was 0.43 for the finetuned-pretrained, 0.036 for the finetuned-shuffled, and 0.035 for the pretrained-shuffled pair.

build upon the same pretrained foundation model. Therefore, a similar attribution analysis result can be expected. When the parameters were “shuffled”, fewer heads appear in the plot. This outcome shows that the XAI method is sensitive to the model parameters, which is expected and required (Adebayo *et al.*, 2018). It varies across different training runs, whereas the finetuned results remain largely similar (as already suggested by the large similarity of full finetuning vs. pretrained). It is noteworthy to stress that on general grounds the plot may still show significant heads due to a coincidental partial alignment of certain randomly initialized heads with directions in feature space that happen to correlate spatially with sequence annotations. Besides, skip connections are not affected by the randomization (Binder *et al.*, 2023). Model parameter randomization tests for XAI evaluation had been introduced by Adebayo *et al.* (2018), and their shortcomings were discussed by Binder *et al.* (2023).

XAI evaluation with residue substitution. XAI methods can be evaluated, e.g. by replacing those input features that have been identified as most relevant for the classification decision of the model with default values or random noise, and by observing the resulting performance drop. In image recognition, this approach has been demonstrated in the form of “pixel flipping” for black and white images, or in the form of pixel substitution with a grey or other color value (Bach *et al.*, 2015). In protein function prediction, the residues in each sequence could be sorted according to the attributed relevance. The top n relevant residues could then be substituted with alanine (cf. alanine scanning mutagenesis; Cunningham and Wells, 1989), while observing the model performance drop in comparison to the substitution of n random residues. We conducted a residue substitution experiment for EC50 L1, where we substituted an increasing number of relevant or random residues with alanine. Random test protein generation was repeated ten times for establishing the random baseline. (The multi-class problem of EC classification on level L1 might lend itself better to this kind of analysis, because there are only six mutually exclusive classes. For the GO classification case, it must be considered that relevance is computed only for one selected class out of the numerous GO terms, while the performance is calculated over all available classes of this multi-label problem.) Table E.4 shows the result of this residue substitution experiment. The model is highly accurate on the original test split. The performance of ProtBert decreases when more and more relevant as well as random residues are substituted with alanine. Performance decays faster when the most relevant residues are substituted, in comparison to a random replacement. Thus, the residues identified with IG are indeed relevant for the accurate classification decision of the model.

Additional XAI results for EC and GO, discussed earlier in Section 5.3, are shown in Supplementary Figures E.2 to E.5.

Relation between homology and XAI. Homology describes the relation between proteins that are deemed as originating from a common ancestor, typically because their sequences or structures are sufficiently similar (or, because an intermediary sequence exists that both sequences resemble) (Pearson, 2013). To study how homology relates to residue-level relevance attributions of an ML model, homology information must be made available per residue. For example, a consensus sequence can be created via MSA, which can be turned into a binary mask of conserved residues present in the respective protein. We have correlated attributions with binary masks of short conserved sequence motifs and with PROSITE patterns. Correlation of attributions with motifs was observed on the embedding level in Figure 5, and, inside of the model for several heads, with motifs and PROSITE patterns in Supplementary Figures E.3 to E.5.

Relation between sequence-structure-function and XAI. According to the “sequence-structure-function” paradigm (Koehler Leman *et al.*, 2023), the amino acid sequence determines the secondary protein structure

Table E.4. Accuracy of ProtBert on EC50 L1 for an increasing number of substituted residues. Either the n most relevant or n random residues of the proteins from the test split were replaced by alanine. Then, the accuracy of the model trained on the original training data was evaluated on these modified test proteins. Performance drops faster when relevant residues are substituted.

n	relevant residues	random residues
0	0.9898	0.9898
1	0.9882	0.9898
2	0.9875	0.9897
4	0.9853	0.9895
8	0.9828	0.9895
16	0.9781	0.9881
32	0.9525	0.9809
64	0.8677	0.9296

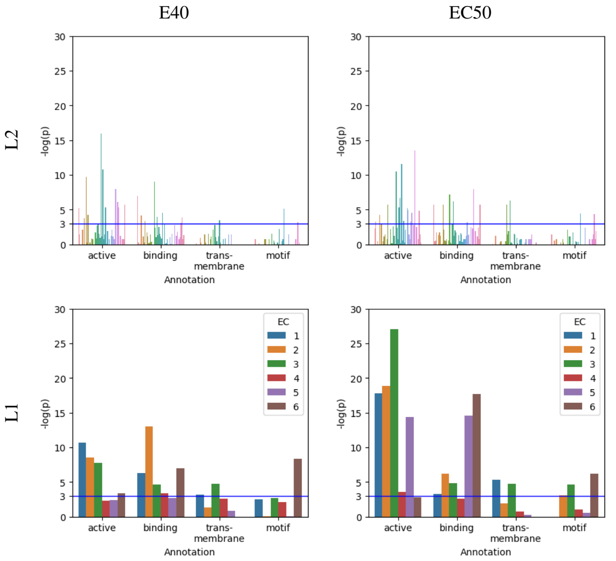

Figure E.2. ProtBert embedding layer attributions were correlated with UniProt sequence annotations, for “EC40” (left) and “EC50” (right), on levels “L1” (bottom) and “L2” (top). Significantly positive relevance-annotation-correlation ($p<0.05$, i.e. above blue line) was observed for “EC40” and “EC50”, on levels “L1” and “L2”, for several enzyme classes (“L1”) and sub-classes (“L2”), and for all annotation types. (Due to the EC hierarchy, more samples were available on level “L1” in comparison to “L2” for each correlation calculation.)

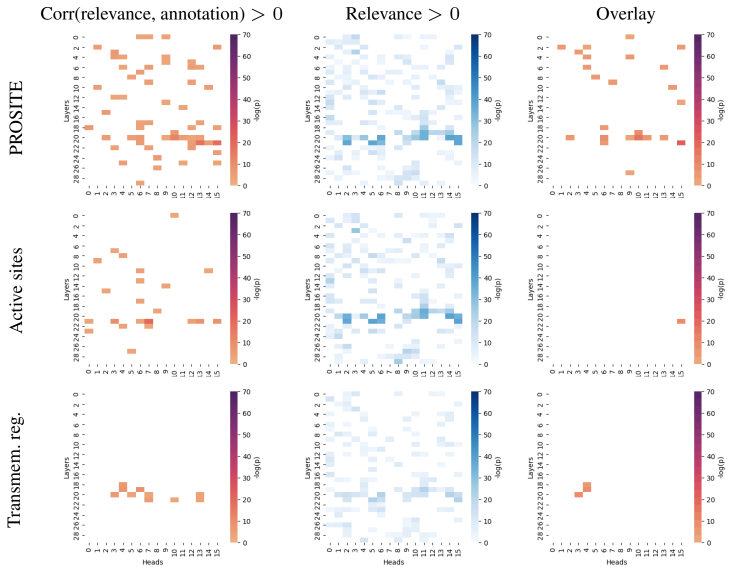

Figure E.3. Inside ProtBert; focus on GO “catalytic activity” (GO:0003824). Left panels: Results of the correlation analysis between relevance attributions indicative for the GO term “catalytic activity” with PROSITE patterns and UniProt annotations as “active sites” and “transmembrane regions” per head and layer (negative logarithm of corrected p-values of Wilcoxon signed-rank tests over correlation coefficients; significance thresholds were overlaid as masks). Center panels: (Negative logarithm of corrected p-values of) Wilcoxon tests inspecting whether the relevance (sum along the sequence) was significantly positive. Right panels: Heads with both a significantly positive attribution-annotation-correlation and a significantly positive relevance (overlay of left and center panels). Only results for corresponding PROSITE patterns (top), UniProt “active sites” (center) and “transmembrane regions” (bottom) are shown. “Binding sites” and “motifs” are ommited, since a significantly positive attribution-annotation-correlation and combined with a positive relevance was not observed in these cases.

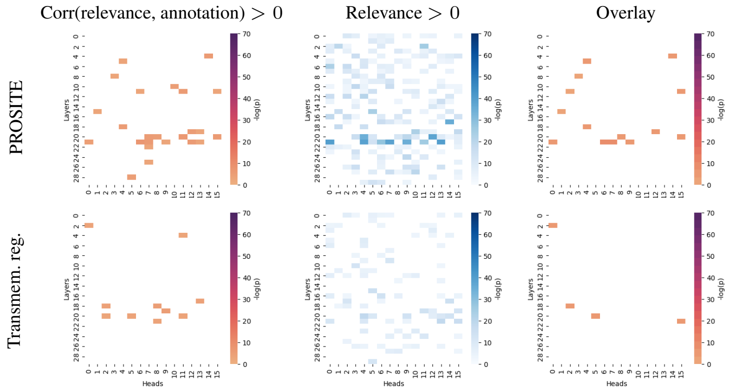

Figure E.4. Inside ProtBert; focus on GO “binding” (GO:0005488). Left: Correlation of relevance for “binding” with corresponding PROSITE patterns (top) or UniProt “transmembrane regions” (bottom) per head and layer. Center: Positive relevance. Right: Heads with both a significantly positive attribution-annotation-correlation and a significantly positive relevance. Results for “active/binding sites” and “motifs” are not shown, given the absence of significantly positive attribution-annotation-correlations in these cases.

(alpha helices, beta sheets etc.) that determines the tertiary protein structure, i.e. 3D shape, that determines the protein function. An analogy can be drawn to the deep ML models that start with the sequence, build up useful representations over multiple layers, to finally classify the protein function. Here, we aimed at inverting this information flow from sequence to function, and attributed relevance for the function prediction model back to the individual residues in the sequences. Interestingly, we observed a high correlation of attributions w.r.t. the GO term “membrane” with annotations as alpha-helical and beta-barrel transmembrane regions (see Figure 3, Figure 5, and Table A.1). Thus, structurally similar sequences apparently resulted in comparable attributions in this case.

Relation to probing and in-silico mutagenesis. The proposed method can provide a complementary view to probing approaches (Vig *et al.*, 2021) as well as to in-silico mutagenesis (‘ISM’; Bromberg and Rost, 2008; Raimondi *et al.*, 2018). ISM and XAI methods have in common that both aim at identifying individual residues that are important for the protein function. ISM uses ML to mimic alanine scanning mutagenesis (Cunningham and Wells, 1989), where single residues are replaced with alanine or other amino acids. Then, the (potentially deleterious) effect of the substitution on the protein function is measured (e.g. binding energy change; see Bromberg and Rost, 2008), respectively predicted with ML. Thereby, residues can be identified that are essential for the protein function (or stability). On the other hand, local XAI methods aim at identifying features (here: residues) that are relevant for the decision of a (protein function prediction) model. XAI post-hoc analyses fall into few general categories. Some XAI methods, including IG, are based on gradients, while other methods are “simulating feature removal” (Covert *et al.*, 2021), e.g. LIME (Ribeiro *et al.*, 2016). In some ways, ISM might be comparable (using the alanine scanning analogy) to feature removal methods where input features are replaced with a given value (alanine) – in principle, PredDiff (Blücher

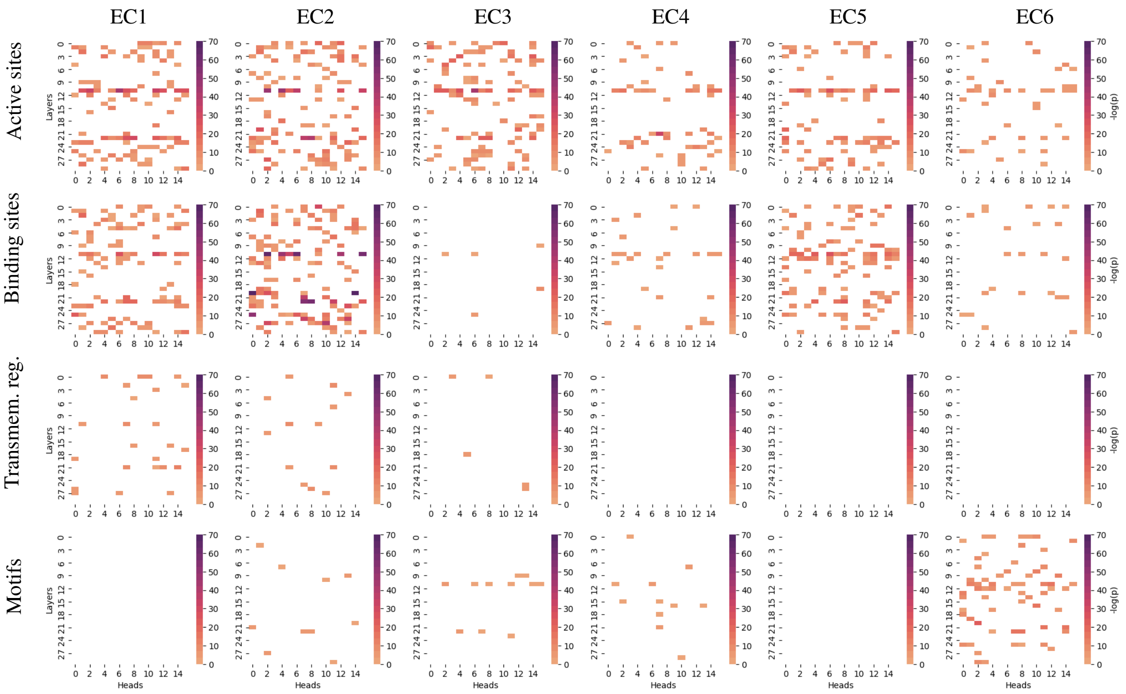

Figure E.5. Inside ProtBert finetuned to EC number classification. Rows: Results of the correlation analysis are presented separately for the different types of annotations (UniProt "active sites", "binding sites", "transmembrane regions", "short sequence motifs"). Columns: Results for the six main EC classes (at level L1). Pixels in the matrix plots represent the 16 transformer heads and 30 layers. Each pixel shows the negative logarithm of the p-value resulting from a Wilcoxon signed-rank test across the coefficients of correlation between relevance attributions and annotation ($p<0.05$; after correction for multiple comparisons and thresholding) overlaid with the mask of those heads/layers that featured a significantly positive relevance too.

et al., 2022) with a constant imputer. Whether ISM or XAI methods are better suited in the context of protein function prediction is hard to say. Both take a different perspective, with ISM potentially placing particular emphasis on the protein stability, and with XAI viewing the problem more indirectly through the "spectacles" of the function prediction model. Finally, a potential shared limitation of ISM and XAI is that both aim merely at identifying individual residues, potentially neglecting their interaction.

Transmembrane regions, hydrophobicity and charge. For a more fine-grained inspection of the case of transmembrane regions presented in Figure 3, we additionally tested if the relevance attributions (to residues of proteins that had been labeled with the GO term "membrane" and annotated with transmembrane regions) correlated with hydrophobic and, respectively, positively charged residues. Hydrophobic amino acids of transmembrane proteins tend to be located in the hydrophobic core of the membrane, while positively charged residues tend to be attracted by the interface of the membrane with the cytoplasma (positive-inside rule; see Vonheijne, 1989; Elazar *et al.*, 2016; Baker *et al.*, 2017). Interestingly, a significant correlation of attributions with residues that were hydrophobic was observed (in particular, if they are situated within a transmembrane region), but not with positively charged residues (see Figure E.6). Apparently, attention of the model, when aiming at inferring if the GO term "membrane" applies to the protein, is particularly drawn to hydrophobic residues buried in the membrane.

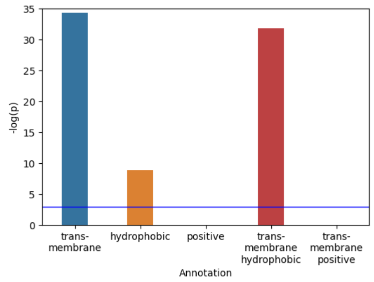

Figure E.6. Relevance attributions to residues of membrane proteins correlate ($p<0.05$; above blue line) with binary masks of hydrophobic residues, in particular if they are located in a transmembrane region, but not with positively charged residues. Thus, the model seems to pay particular attention to hydrophobic residues buried in the membrane when inferring whether the GO term "membrane" applies to the protein.

References

Adebayo, J. *et al.* (2018). Sanity checks for saliency maps. *Adv. neural inf. process. syst.*, **31**.

Ashburner, M. *et al.* (2000). Gene Ontology: tool for the unification of biology. *Nat. genet.*, **25**(1), 25–29.

Bach, S. *et al.* (2015). On pixel-wise explanations for non-linear classifier decisions by layer-wise relevance propagation. *PLoS ONE*, **10**(7).

Baker, J. A. *et al.* (2017). Charged residues next to transmembrane regions revisited: "Positive-inside rule" is complemented by the "negative inside depletion/outside enrichment rule". *BMC Biology*, **15**(1), 1–29.

Bernhofer, M. *et al.* (2021). PredictProtein - Predicting Protein Structure and Function for 29 Years. *Nucleic Acids Res.*, **49**(W1), W535–W540.

Binder, A. *et al.* (2023). Shortcomings of top-down randomization-based sanity checks for evaluations of deep neural network explanations. In *Proc. IEEE/CVF CVPR*, pages 16143–16152, Vancouver, BC, Canada.

Blücher, S. *et al.* (2022). PredDiff: Explanations and interactions from conditional expectations. *Artificial Intelligence*, **312**, 103774.

Bromberg, Y. and Rost, B. (2008). Comprehensive in silico mutagenesis highlights functionally important residues in proteins. *Bioinformatics*,

24(16), i207–i212.

Clark, W. T. and Radivojac, P. (2013). Information-theoretic evaluation of predicted ontological annotations. *Bioinformatics*, **29**(13), i53–i61.

Consortium, G. O. (2020a). The Gene Ontology resource: enriching a GOld mine. *Nucleic Acids Res.*, **49**(D1), D325–D334.

Consortium, U. (2020b). UniProt: the universal protein knowledgebase in 2021. *Nucleic Acids Res.*, **49**(D1), D480–D489.

Covert, I. *et al.* (2021). Explaining by Removing: A Unified Framework for Model Explanation. *J. Mach. Learn. Res.*, **22**(209), 1–90.

Cunningham, B. C. and Wells, J. A. (1989). High-resolution epitope mapping of hGH-receptor interactions by alanine-scanning mutagenesis. *Science*, **244**(4908), 1081–1085.

Dalkiran, A. *et al.* (2018). ECPred: a tool for the prediction of the enzymatic functions of protein sequences based on the EC nomenclature. *BMC Bioinform.*, **19**(1), 334.

Devlin, J. *et al.* (2018). BERT: Pre-training of deep bidirectional transformers for language understanding. *arXiv:1810.04805*.

Elazar, A. *et al.* (2016). Interplay between hydrophobicity and the positive-inside rule in determining membrane-protein topology. *PNAS*, **113**(37), 10340–10345.

Elnaggar, A. *et al.* (2022). ProtTrans: Toward Understanding the Language of Life Through Self-Supervised Learning. *IEEE Trans. Pattern Anal. Mach. Intell.*, **44**(10), 7112–27.

Jumper, J. *et al.* (2021). Highly accurate protein structure prediction with AlphaFold. *Nature*, **596**(7873), 583–589.

Kingma, D. P. and Ba, J. (2015). Adam: A method for stochastic optimization. In *3rd ICLR*, San Diego.

Koehler Leman, J. *et al.* (2023). Sequence-structure-function relationships in the microbial protein universe. *Nature communications*, **14**(1), 2351.

Kokhlikyan, N. *et al.* (2020). Captum: A unified and generic model interpretability library for PyTorch. *arXiv:2009.07896*.

Kulmanov, M. and Hoehndorf, R. (2019). DeepGOPlus: improved protein function prediction from sequence. *Bioinformatics*, **36**(2), 422–429.

Kulmanov, M. and Hoehndorf, R. (2022). DeepGOZero: improving protein function prediction from sequence and zero-shot learning based on ontology axioms. *Bioinformatics*, **38**(Supp. 1), i238–i245.

Kulmanov, M. *et al.* (2017). DeepGO: predicting protein functions from sequence and interactions using a deep ontology-aware classifier. *Bioinformatics*, **34**(4), 660–668.

Li, Y. *et al.* (2018). DEEPre: sequence-based enzyme EC number prediction by deep learning. *Bioinformatics*, **34**(5), 760–769.

Lin, Z. *et al.* (2023). Evolutionary-scale prediction of atomic-level protein structure with a language model. *Science*, **379**(6637), 1123–30.

Littmann, M. *et al.* (2021). Embeddings from deep learning transfer GO annotations beyond homology. *Sci. rep.*, **11**(1), 1–14.

McDonald, A. G. *et al.* (2008). ExplorEnz: the primary source of the IUBMB enzyme list. *Nucleic Acids Res.*, **37**(S. 1), D593–D597.

Olenyi, T. *et al.* (2023). LambdaPP: Fast and accessible protein-specific phenotype predictions. *Protein Science*, **32**(1), e4524.

Paszke, A. *et al.* (2019). PyTorch: An Imperative Style, High-Performance Deep Learning Library. In H. Wallach, H. Larochelle, A. Beygelzimer, F. d'Alché-Buc, E. Fox, and R. Garnett, editors, *Adv. NeurIPS 32*, pages 8024–35. Curran Assoc., Vancouver, Canada.

Pearson, W. R. (2013). An Introduction to Sequence Similarity ("Homology") Searching. *CP Bioinformatics*, **42**(1), 3.1.1–3.1.8.

Raffel, C. *et al.* (2020). Exploring the Limits of Transfer Learning with a Unified Text-to-Text Transformer. *J. Mach. Learn. Res.*, **21**(140), 1–67.

Raimondi, D. *et al.* (2018). Large-scale in-silico statistical mutagenesis analysis sheds light on the deleteriousness landscape of the human proteome. *Sci. Rep.*, **8**(1), 16980.

Rao, R. *et al.* (2019). Evaluating Protein Transfer Learning with TAPE. In H. Wallach, H. Larochelle, A. Beygelzimer, F. d'Alché-Buc, E. Fox, and R. Garnett, editors, *Adv. Neural Inf. Process. Syst.*, volume 32, Vancouver, Canada. Curran Assoc.

Reimers, N. and Gurevych, I. (2019). Sentence-BERT: Sentence Embeddings using Siamese BERT-Networks. In *Proc. '19 EMNLP*, Hong Kong, China. ACL.

Ribeiro, M. T. *et al.* (2016). "Why Should I Trust You?": Explaining the Predictions of Any Classifier. In *Proc. 22nd ACM SIGKDD*, KDD '16, page 1135–1144, New York, NY, USA. ACM.

Seabold, S. and Perktold, J. (2010). Statsmodels: Econometric and statistical modeling with Python. In *Proc. SciPy 2010*, volume 57, pages 10–25080. Austin, Texas.

Sigrist, C. J. *et al.* (2012). New and continuing developments at PROSITE. *Nucleic Acids Res.*, **41**(D1), D344–D347.

Steinegger, M. and Söding, J. (2018). Clustering huge protein sequence sets in linear time. *Nat. Commun.*, **9**(1), 1–8.

Steinegger, M. *et al.* (2019). Protein-level assembly increases protein sequence recovery from metagenomic samples manyfold. *Nat. Methods*, **16**(7), 603–606.

Strodthoff, N. *et al.* (2020). UDSMProt: universal deep sequence models for protein classification. *Bioinformatics*, **36**(8), 2401–9.

Sundararajan, M. *et al.* (2017). Axiomatic Attribution for Deep Networks. In D. Precup and Y. W. Teh, editors, *Proc. 34th ICML*, volume 70 of *Proc. Mach. Learn. Res.*, pages 3319–28, Sydney, Australia. PMLR.

Suzek, B. E. *et al.* (2014). UniRef clusters: a comprehensive and scalable alternative for improving sequence similarity searches. *Bioinformatics*, **31**(6), 926–932.

Vig, J. *et al.* (2021). BERTology Meets Biology: Interpreting Attention in Protein Language Models. In *ICLR 2021*, Virtual Event.

Virtanen, P. *et al.* (2020). SciPy 1.0: Fundamental Algorithms for Scientific Computing in Python. *Nat. Methods*, **17**, 261–272.

Vonheijne, G. (1989). Control of topology and mode of assembly of a polytopic membrane protein by positively charged residues. *Nature*, **341**(6241), 456–458.

Webb, E. C. *et al.* (1992). *Enzyme nomenclature 1992. Recommendations of the Nomenclature Committee of the IUBMB on the Nomenclature and Classification of Enzymes.* Number Ed. 6. Academic Press.

You, R. *et al.* (2018a). DeepText2GO: Improving large-scale protein function prediction with deep semantic text representation. *Methods*, **145**, 82–90.

You, R. *et al.* (2018b). GOLabeler: improving sequence-based large-scale protein function prediction by learning to rank. *Bioinformatics*, **34**(14), 2465–73.

You, R. *et al.* (2021). DeepGraphGO: graph neural network for large-scale, multispecies protein function prediction. *Bioinformatics*, **37**(1), i262–71.

Yu, T. *et al.* (2023). Enzyme function prediction using contrastive learning. *Science*, **379**(6639), 1358–63.

Zhou, N. *et al.* (2019). The CAFA challenge reports improved protein function prediction and new functional annotations for hundreds of genes through experimental screens. *Genome biol.*, **20**(1), 1–23.

Zou, Z. *et al.* (2019). mlDEEPre: Multi-Functional Enzyme Function Prediction With Hierarchical Multi-Label Deep Learning. *Front. Genet.*, **9**, 714.
